# The Effects of Forming CeFe_2_ on Phase Structure and Magnetic Properties in Ce-Rich Nd-Ce-Fe-B Permanent Magnetic Materials

**DOI:** 10.3390/ma14206070

**Published:** 2021-10-14

**Authors:** Min Huang, Zhiqiang Qiu, Fang Wang, Hubin Luo, Changping Yang, Jian Zhang

**Affiliations:** 1 CAS Key Laboratory of Magnetic Materials and Devices, Ningbo Institute of Materials Technology and Engineering, Chinese Academy of Sciences, Ningbo 315201, China; huangmin@nimte.ac.cn (M.H.); qiuzhiqiang@nimte.ac.cn (Z.Q.); luohubin@nimte.ac.cn (H.L.); 2 Zhejiang Province Key Laboratory of Magnetic Materials and Application Technology, Ningbo Institute of Materials Technology and Engineering, Chinese Academy of Sciences, Ningbo 315201, China; 3 Faculty of Physics and Electronic Science, Hubei University, Wuhan 430062, China; 4 School of Materials and Chemical Engineering, Ningbo University of Technology, Ningbo 315211, China; wangfangch@nbut.edu.cn

**Keywords:** Nd-Ce-Fe-B magnets, kink, CeFe_2_ phase, coercivity

## Abstract

The decomposition of the Nd-Ce-Fe-B phase to form CeFe_2_ has been usually believed to have an important positive effect on the magnetic properties of Nd-Ce-Fe-B permanent magnetic materials. In this work, a new decomposition process of the Nd-Ce-Fe-B phase on the formation of the CeFe_2_ phase was observed to play a negative role in its magnetic properties. It is demonstrated that the Nd-Ce-Fe-B phase decomposes into non-magnetic CeFe_2_, accompanied by the precipitation of Fe soft-phase. The kinks usually occurring in the demagnetization curves of Ce-rich Nd-Ce-Fe-B magnets have been determined to be related to the Fe soft-phase. Instead of using CeFe_2_ as a grain-boundary phase, another Ce-Cu boundary phase has been explored to efficiently improve the coercivity of Ce-rich Nd-Ce-Fe-B magnets, provided that the Ce-Cu boundary phase has an appropriate Ce to Cu ratio. The present results contribute to the mechanism comprehension and high-performance design of Nd-Ce-Fe-B permanent magnetic materials.

## 1. Introduction

Among permanent magnets, Nd_2_Fe_14_B-based magnets have been widely used in many fields due to their highest maximum energy product (BH)_max_. In particular, they have been used in energy-related technologies such as hybrid cars and gearless wind turbines [1,2]. With the expansion of the Nd-Fe-B industry, non-renewable rare earth resources have become fewer and fewer, leading to the price increase of critical rare earth metals (Nd, Pr, Dy, etc.). Therefore, the cost of raw materials has put considerable pressure on the Nd-Fe-B industry and its downstream applications [3]. In addition, high-abundant and low-cost La and Ce metals separated from Nd and Pr are often overstocked over a long period of time. The efficient use of La and Ce not only reduces the raw material cost of the Nd-Fe-B industry, but also balances the utilization of rare resources and solves environmental pollution problems [4,5,6]. It was reported that Ce_2_Fe_14_B phase is more stable than La_2_Fe_14_B phase [7], thus, extensive works have been carried out on the substitution for Nd using the high-abundant Ce to develop low-cost permanent magnets [8,9,10,11]. 

However, the Nd-Ce-Fe-B magnets have unique features that are distinct from Nd-Fe-B, one of which is the formation of CeFe_2_ phase. Previous works have demonstrated that the CeFe_2_ phase is inevitable in Nd-Ce-Fe-B, with the Ce content exceeding the critical value of 21.5% (at.%) [12,13]. Particularly, the mass fraction of the CeFe_2_ phase can even exceed the amount of other RE-rich phase with a further raised Ce substitution level [13]. That is, the CeFe_2_ phase progressively evolves as the dominant intergranular phase in Ce-rich magnets [13,14,15]. Therefore, understanding the role of CeFe_2_ phase in Nd-Ce-Fe-B magnetic properties has become a fundamental concern. Most studies confirmed the positive effects of the CeFe_2_ phase. During the liquid-phase-sintering process, the CeFe_2_ phase with a lower melting point than the sintering temperature is beneficial for promoting the formation of the homogenous grain boundary phase. The melted CeFe_2_ phase plays a similar role as the RE-rich phase to facilitate the liquid flowing and 2:14:1 particles rearrangement. The magnets exhibit more uniform and continuous distribution of intergranular phases surrounding the 2:14:1 matrix phase grains, which improves the coercivity of the Nd-Ce-Fe-B magnets [16,17,18]. Additionally, it was also reported that the formation of CeFe_2_ phase would result in an increase in the amount of metallic RE-rich phases [12], which eventually led to a thickened grain boundary and isolated the adjacent hard magnetic grains, contributing to the enhancement of coercivity [19]. Nevertheless, the CeFe_2_ phase has a Curie temperature below room temperature. The presence of paramagnetic CeFe_2_ will more or less decrease the magnetization of Nd-Ce-Fe-B magnets. Furthermore, in recent investigations, whether in films, ribbons, or sintered Ce-rich Nd-Ce-Fe-B magnets, kinks usually occurred in the hysteresis loops [20,21], inducing the reduced remanence and maximum magnetic energy product (which obviously deteriorates the magnetic properties). However, the reasons for the formation of kinks in the hysteresis loops of Ce-rich Nd-Ce-Fe-B are still unclear.

Recently, investigations on the preparation of Nd-Fe-B permanent magnetic film by physical vapor deposition (PVD) technology have aroused wide interest [22,23,24,25,26]. Thin film is usually used as a model case for bulk magnets due to the readily controlled microstructure [22,23,24,25,26], which benefits the analysis of the performance mechanism in bulk magnets from an experimental point of view. In this paper, the phase structure and magnetic properties of Nd-Ce-Fe-B films have been studied. A new mechanism for the deterioration of magnetic properties by forming the CeFe_2_ phase is discovered. It is demonstrated that the forming CeFe_2_ phase in Ce-rich Nd-Ce-Fe-B permanent magnetic materials will lead to the precipitation of α-Fe, which results in the formation of obvious kinks in the hysteresis loops. Furthermore, very high coercivity is acquired in Ce-rich Nd-Ce-Fe-B films by using the Ce-Cu grain boundary diffusion layers with proper Ce to Cu ratio.

## 2. Materials and Methods

**Figure 1:**
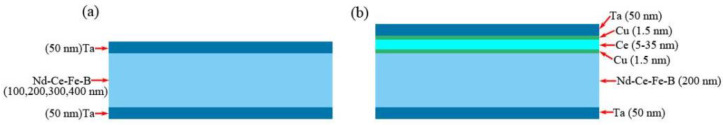
The schematic diagram of multilayer films prepared in this work. (**a**) Ta (50 nm)/Nd-Ce-Fe-B (t_NdCeFeB_ nm)/Ta (50 nm) film. (**b**) Ta (50 nm)/Nd-Ce-Fe-B (200 nm)/Cu (1.5 nm)/Ce (t_Ce_ nm)/Cu (1.5 nm)/Ta (50 nm) film.

Figure 1 is the schematic diagram of Ta/Nd-Ce-Fe-B/Ta (denoted as Nd-Ce-Fe-B (t_Nd-Ce-Fe-B_ nm)) and Ta/Nd-Ce-Fe-B/Cu/Ce/Cu/Ta (denoted as Nd-Ce-Fe-B/Cu/Ce (t_Ce_ nm)/Cu) films fabricated by magnetron sputtering system with a base pressure of 2.2 × 10^−7^ Pa. The Nd-Ce-Fe-B films were deposited on thermally oxidized silicon substrates with a Ta underlayer. The Ta underlayer and coverlayer of 50 nm were prepared to suppress oxidation of the Nd-Ce-Fe-B films. The purities of commercial Ta, Cu and Ce targets are 99.95%. The Nd-Ce-Fe-B layer was sputtered from a target of size Ф 60 × 6 mm^2^ and of nominal composition Nd_6.19_Ce_7.98_Fe_75.48_B_10.35_ (at.%). All the targets were mounted on Cu or stainless-steel backing plates. The Ta layer was deposited at the temperature of 300 °C and 120 w DC power. The Cu and Ce layer were deposited in the cooling process with the temperature lower than 250 °C, and the corresponding AC powers were 15 w and 60 w, respectively. The Nd-Ce-Fe-B layer was deposited with a 140 w DC power. In Nd-Ce-Fe-B (400 nm) films, different deposition temperatures of Nd-Ce-Fe-B layer were chosen as 550, 600, 625 and 650 °C for comparison. For Nd-Ce-Fe-B (t_Nd-Ce-Fe-B_ nm) films, the deposition temperature keeps at 600 °C for Nd-Ce-Fe-B layer, and the nominal thickness of the Nd-Ce-Fe-B layer (t_Nd-Ce-Fe-B_) was chosen as 100, 200, and 300 nm. For Nd-Ce-Fe-B /Cu/Ce (t_Ce_ nm)/Cu films, the deposition temperature for Nd-Ce-Fe-B layer was maintained at 600 °C. The thicknesses of Nd-Ce-Fe-B and Cu layers are 200 nm and 1.5 nm, respectively, while the thickness of Ce layer (t_Ce_ nm) was varied in the range from 5 to 35 nm. All above thicknesses were calculated as the product of the deposition rate and the deposition time. The entire deposition process was carried out under high purity argon flow at 0.8 Pa. And the as-deposited films were subsequently annealed in situ at the temperature of 650 °C for 30 min.

The constituent phases were characterized by X-ray diffraction (XRD, Bruker, Karlsruhe, Germany) using Cu Kα radiation. The hysteresis loops along the perpendicular directions were measured by the physical property measurement system (PPMS, Quantum Design company, San Diego, CA, USA).

## 3. Results and Discussion

**Figure 2:**
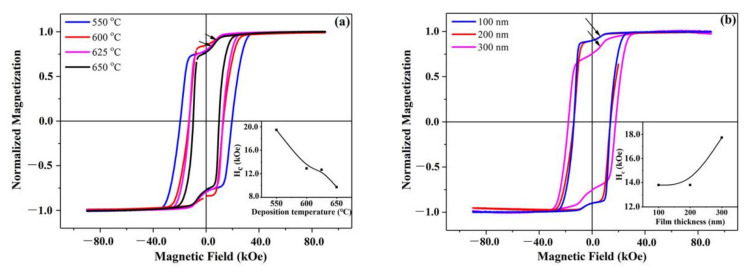
(**a**) Perpendicular magnetic hysteresis loops of Ta (50 nm) /Nd_6.19_Ce_7.98_Fe_75.48_B_10.35_ (400 nm)/Ta (50 nm) films deposited at different temperatures with the corresponding coercivity values in the inset. (**b**) Perpendicular magnetic hysteresis loops of Ta(50 nm)/Nd_6.19_Ce_7.98_Fe_75.48_B_10.35_(t_Nd-Ce-Fe-B_ nm)/Ta (50 nm) films with different Nd-Ce-Fe-B layer thicknesses (t_Nd-Ce-Fe-B_ = 100, 200, 300), and the corresponding coercivity values were shown in the inset.

Figure 2a shows the room temperature demagnetization curves for Nd-Ce-Fe-B (400 nm) films deposited at different temperatures, which was measured in an applied field of ±90 kOe perpendicular to the film plane. Among these four films, obvious kinks marked by the arrows occur in the hysteresis loops. In the films deposited at 550, 625, and 650 °C, the kinks are worse than that in the film deposited at 600 °C. The coercivity values of the films as a function of deposition temperature are given in the inset of Figure 2a. With the deposition temperature increasing, the coercivity decreases. A very high coercivity of about 19.4 kOe is obtained in the film deposited at 550 °C. 

Figure 2b shows the room temperature demagnetization curves of Nd-Ce-Fe-B (t_Nd-Ce-Fe-B_ nm) films (t_Nd-Ce-Fe-B_=100, 200, 300) deposited at 600 °C, which was also measured in an applied field of ±90 kOe perpendicular to the film plane. There is also the appearance of kinks marked by the arrows in the hysteresis loops of these films. The kink of the Nd-Ce-Fe-B (300 nm) film, however, is worse than that of the other two films. The coercivity values of the films as a function of film thickness are given in the inset. As the film thickness increases from 100 nm to 300 nm, the coercivity rises from 13.8 kOe to 17.7 kOe.

**Figure 3:**
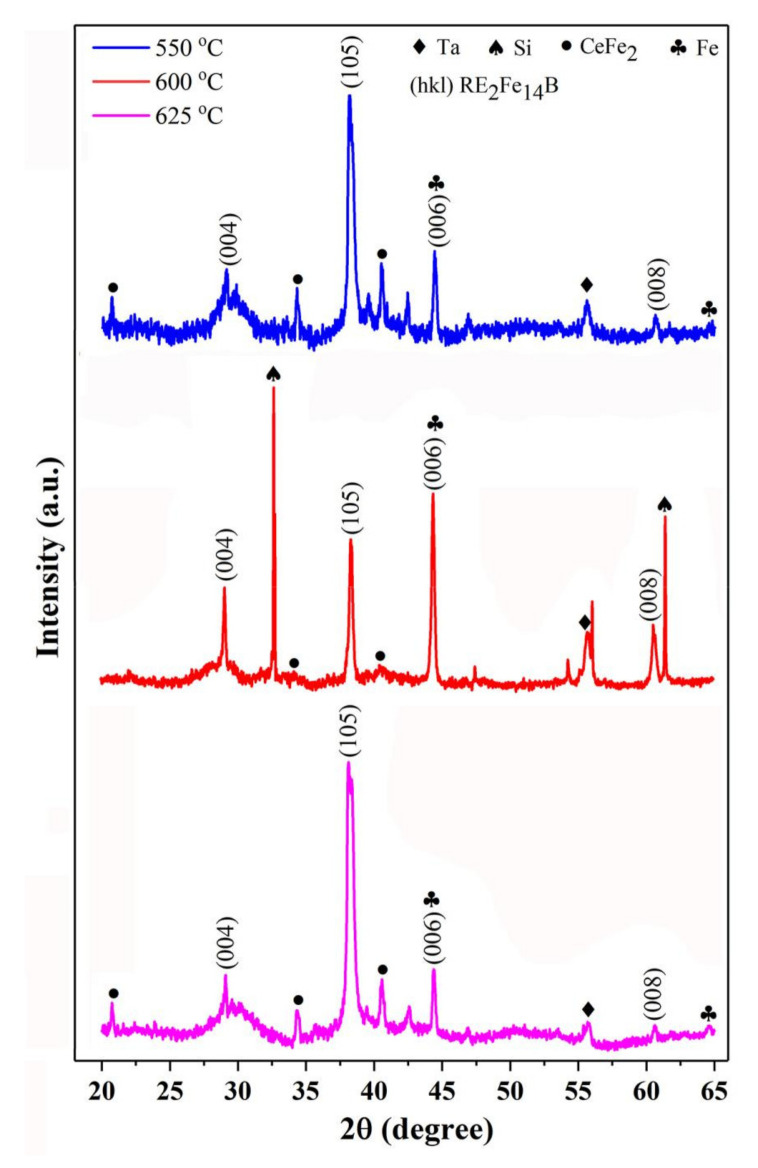
XRD patterns of Ta (50 nm)/Nd_6.19_Ce_7.98_Fe_75.48_B_10.35_ (400 nm)/Ta (50 nm) films deposited at different temperatures.

**Figure 4:**
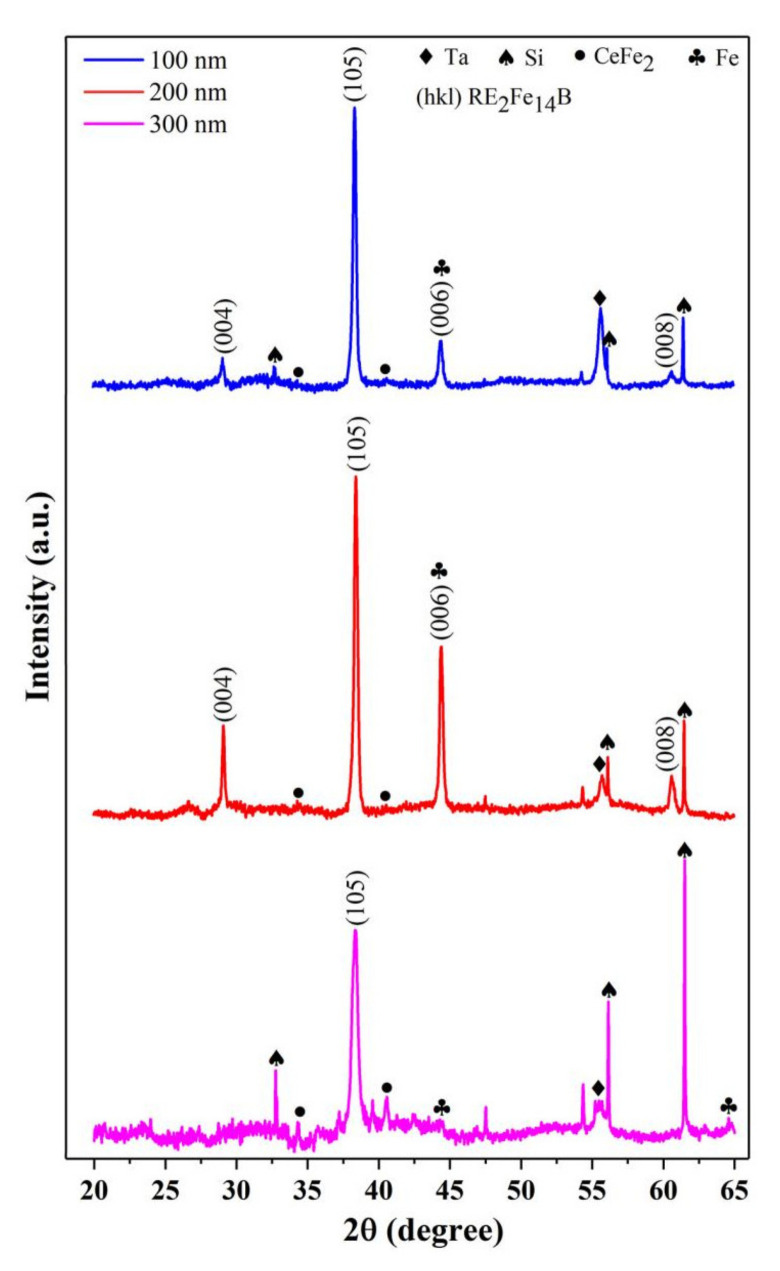
XRD patterns of Ta (50 nm)/Nd_6.19_Ce_7.98_Fe_75.48_B_10.35_ (t_Nd-Ce-Fe-B_ nm)/Ta (50 nm) films (t_Nd-Ce-Fe-B_ = 100, 200, 300) deposited at 600 °C.

In the hysteresis loops of the films shown in Figure 2, some kinks are worse and some kinks are slight. The kinks are also found in previously reported Ce-rich Nd-Ce-Fe-B sintered magnets [20,21]. Apparently, the existence of a kink can deteriorate the magnetic properties. However, the reasons for the formation of kinks are still not clear. In order to elucidate the reasons behind this, the phase structures in the films were analyzed by XRD. Figure 3 gives the X-ray diffraction patterns for the Nd-Ce-Fe-B (400 nm) films deposited at different temperatures. Partial diffraction peaks of Ta and Si are detected owing to the buffer/covering layer and substrate. Both of them are nonmagnetic phases and do not contribute to the kinks in the hysteresis loops. As shown in Figure 3, prominent (105) and (006) characterization diffraction peaks of (Nd,Ce)_2_Fe_14_B are obtained for the Nd-Ce-Fe-B (400 nm) films, indicating that the gains of the Nd-Ce-Fe-B phase are well textured and their easy magnetization axis (c-axis) is perpendicular to the film plane. The nominal composition of Nd-Ce-Fe-B film is Nd_6.19_Ce_7.98_Fe_75.48_B_10.35_ (at.%), and Ce accounts for 56.3% (at.%) of the total rare earths, and is well above the critical value 21.5% (at.%) [12,13]. Therefore, the formation of the CeFe_2_ phase is inevitable, which is consistent with the observed CeFe_2_ peaks in Figure 3. By combining Figure 2a with Figure 3, it is found that the kinks in the hysteresis loops for the films deposited at 550 and 625 °C are worse, and more CeFe_2_ phase is detected in these two films, while the kink in the hysteresis loop for the film deposited at 600 °C is slight and, correspondingly, its CeFe_2_ phase is less. Figure 4 shows the XRD patterns for Nd-Ce-Fe-B (t_Nd-Ce-Fe-B_ nm) films with different thicknesses (t_Nd-Ce-Fe-B_ =100, 200, 300) deposited at 600 °C. Similar phase structure was observed in Figure 4. By combining Figure 2b with Figure 4, it is also found that the slight kinks occur in the hysteresis loops for Nd-Ce-Fe-B (100 nm) and Nd-Ce-Fe-B (200 nm) films, and the amount of CeFe_2_ phase is less in these two films, while the worse kink emerges in the hysteresis loop for Nd-Ce-Fe-B (300 nm) film, and correspondingly, its amount of CeFe_2_ phase is more. These observations indicates that the increasing CeFe_2_ phase in Nd-Ce-Fe-B films should worsen kinks in the hysteresis loops.

However, the CeFe_2_ phase is paramagnetic at room temperature, (its Curie temperature (Tc) is about 227 K [27]). The paramagnetic CeFe_2_ phase itself makes no contribution to the kinks in the hysteresis loops. Previous investigations have shown that the formation of CeFe_2_ phase is due to the fact that the (Nd,Ce)_2_Fe_14_B phase become unstable with raising the Ce substitution level [28]. According to Figure 3 and Figure 4, it is worth noting that less α-Fe phase is detected when the CeFe_2_ is less in Nd-Ce-Fe-B films. The obvious diffraction peak of α-Fe at 2θ = 64.6° is observed only when the amount of CeFe_2_ phase is relatively high. It is confirmed that the CeFe_2_ phase will promote the precipitation of α-Fe. Therefore, we manifest a new decomposition process for the Ce-rich (Nd,Ce)_2_Fe_14_B phase. The unstable Ce-rich (Nd,Ce)_2_Fe_14_B phase is actually easily decomposed into not only CeFe_2_ but also the α-Fe phase. The α-Fe phase is well soft-magnetic. During the demagnetization process, the soft α-Fe phase will firstly be reversed, leading to the occurrence of kinks in the hysteresis loops. The soft-magnetic α-Fe induced by forming the CeFe_2_ phase is the direct reason for the worse kinks observed in the hysteresis loops of Ce-rich Nd-Ce-Fe-B magnets.

According to the hysteresis loops in Figure 2, the magnetization is mainly a result of the soft-magnetic α-Fe and hard-magnetic (Nd,Ce)_2_Fe_14_B phases. The decrease in magnetization at the kink marked by arrows is due to the α-Fe phase, and the remaining reduction in magnetization is attributed to the (Nd,Ce)_2_Fe_14_B phase. If x and y represent the content of α-Fe and (Nd,Ce)_2_Fe_14_B, respectively, they will satisfy the following equation:

$$\frac{M_{s}\left(\mathrm{Fe}\right)\;*\;x}{M_{s\;}\left({\left(\mathrm{Nd},\mathrm{Ce}\right)}_{2\;}{\mathrm{Fe}}_{14}B\right)\;*\;y}=\frac{\Delta M_{d\;}\left(\mathrm{Fe}\right)}{\Delta M_{d\;}\left({\left(\mathrm{Nd},\mathrm{Ce}\right)}_{2}{\mathrm{Fe}}_{14}B\right)}$$

where M_s_(Fe) and M_s_((Nd,Ce)_2_Fe_14_B) are the saturation magnetization of α-Fe and (Nd,Ce)_2_Fe_14_B, respectively. ΔM_d_(Fe) and ΔM_d_((Nd,Ce)_2_Fe_14_B) are the decrease in magnetization resulting from α-Fe and (Nd,Ce)_2_Fe_14_B, respectively. The saturation magnetization can be obtained according to the literature [29], while the decrease in magnetization caused by different phases can be directly acquired from Figure 2. After calculation, the results of x/y in different preparation condition are listed in Table 1. In the films deposited at 550, 625, and 650 °C, the amount of α-Fe is higher than that in the film deposited at 600 °C. Similarly, in the Nd-Ce-Fe-B (300 nm) film, the amount of α-Fe is higher than that of the Nd-Ce-Fe-B (100 nm) and Nd-Ce-Fe-B (200 nm) films. The quantitative Fe contents calculated from the hysteresis loops are in perfect agreement with the XRD results (see Figure 3 and Figure 4), which further confirmed a certain correlation between the kinks in hysteresis and the α-Fe phase. Unfortunately, the Rietveld analysis of X-ray data did not provide good quantitative results since the peaks of (006) and α-Fe in XRD patterns overlap in the 45° area.

The occurrence of kinks in the hysteresis loops has also been observed previously in film, ribbon, and sintered Nd-Ce-Fe-B magnets [20,21]. However, their formation mechanism is still unclear. Our study indicates that it originated from the soft α-Fe phase owing to the formation of the CeFe_2_ phase. Usually, the forming CeFe_2_ has been believed to have a positive effect and improve effectively the coercivity of Nd-Ce-Fe-B magnets. Our study, however, indicates that the forming CeFe_2_ will have a negative effect on the magnetic properties of Nd-Ce-Fe-B magnets. It will induce harmful kinks in the hysteresis loop. Additionally, it will lead to the precipitation of α-Fe, which is unhelpful for the improvement of the coercivity. Our study is of significance for the mechanism understanding and high-performance designing of Ce-rich Nd-Ce-Fe-B magnets.

It should be mentioned that it is the varying degrees of worsen kinks in the hysteresis loops induced by different film fabrication conditions (see Figure 2) that allows us to find the new decomposition process of the (Nd,Ce)_2_Fe_14_B phase. With proper deposition temperature at 600 °C, the (Nd,Ce)_2_Fe_14_B phase will not be easily decomposed. However, a higher deposition temperature should accelerate the decomposition of (Nd,Ce)_2_Fe_14_B phase, leading to the occurrence of worse kink in the hysteresis loops. It is likely that for thinner films (100 nm and 200 nm), a greater amount of (Nd,Ce)_2_Fe_14_B phases are epitaxially grown and stabilized upon the substrate, which causes the less serious kinks in the hysteresis loops. For the thicker film, the CeFe_2_ phase will tend to be formed [30], resulting in worse kinks occurring in the hysteresis loops.

**Figure 5:**
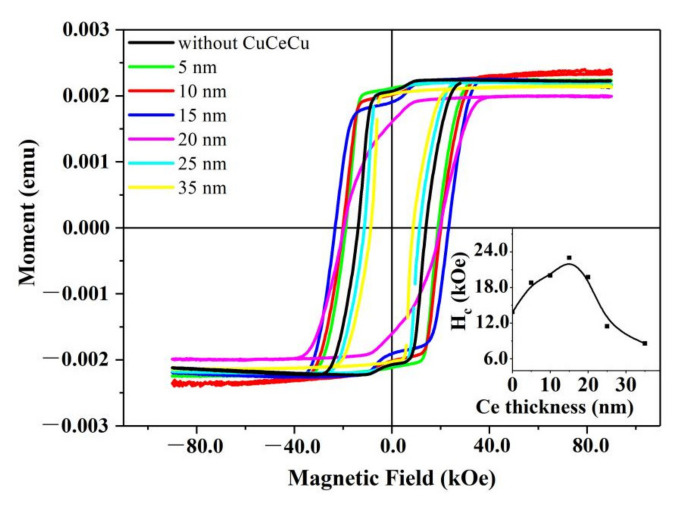
Perpendicular magnetic hysteresis loops of Ta(50 nm)/Nd_6.19_Ce_7.98_Fe_75.48_B_10.35_ (200 nm)/Cu(1.5 nm)/Ce(t_Ce_ nm)/Cu(1.5 nm)/Ta(50 nm) films (t_Ce_ = 5, 10, 15, 20, 25, 35) and Ta(50 nm) Nd_6.19_Ce_7.98_Fe_75.48_B_10.35_ (200 nm)/Ta(50 nm) film (named without CuCeCu), and the corresponding coercivity values were shown in the inset.

The grain boundaries phase, favoring the wetting and isolating of the adjacent hard magnetic grains, plays a key role in improving the coercivity of Nd_2_Fe_14_B-based magnets. Usually, the paramagnetic CeFe_2_ is distributed among the grain boundaries and is beneficial for the improvement of coercivity in Nd-Ce-Fe-B magnets [16,17,18,31]. However, according to the study above, the forming CeFe_2_ phase will promote the precipitation of α-Fe, which will definitely deteriorate its magnetic properties. Thus, the CeFe_2_ phase should not be too great in Ce-rich Nd-Ce-Fe-B magnets. In order to further improve the coercivity of Ce-rich Nd-Ce-Fe-B films, other grain boundary phase such as low melting Ce-Cu phase is explored. The Ce-Cu boundary phase with different composition is introduced by designing the Ce/Cu diffusion layer. Figure 5 shows the room temperature demagnetization curves for the Nd-Ce-Fe-B/Cu/Ce (t_Ce_ nm)/Cu films. It is worth noting that the relatively low Ce to Cu ratio will not make the kink worse, but it can improve the coercivity obviously, indicating the validity of adding Ce-Cu for improving magnetic properties. The coercivity values as a function of the Ce layer thickness in the films are indicated in the inset of Figure 5. With the thickness of the Ce layer increasing, coercivity firstly increases, reaching a maximum, then decreases. The maximum coercivity is acquired with the thickness of Ce layer of 15 nm. In Nd-Ce-Fe-B /Cu/Ce (t_Ce_ nm)/Cu films, the added Cu/Ce/Cu layer will react to form the eutectic phase and have the possibility to melt after annealing at 650 °C for 30 min. It will easily penetrate into the grain boundaries and optimize the microstructure through wetting or isolating the 2:14:1 main phases, leading to the enhancement of coercivity [19]. For the Nd-Ce-Fe-B/Cu/Ce(15 nm)/Cu film, the atom ratio of Ce to Cu is the nearest to 72/28 (at.%), which is the eutectic point (lowest melting point) in the Ce-Cu binary phase diagram. Under such composition circumstances, the total Ce and Cu may form the completely melting Ce-Cu eutectic phase, easily and completely diffusing into the grain boundary. Therefore, an extremely high coercivity of about 23 kOe is achieved. Compared to the initial Nd-Ce-Fe-B film without Cu/Ce/Cu diffusion layer, the coercivities of Nd-Ce-Fe-B /Cu/Ce(5–15 nm)/Cu films are much increased by 4.9–9.1 kOe without introducing more worse kinks in the hysteresis loops. With the further increasing Ce content, extra Ce in the Cu/Ce/Cu layer may substitute Nd in the 2:14:1 phase, leading to the reduced coercivity. When the thickness of the Ce layer reached 25 nm or more, the coercivity is even much smaller than that without the Cu/Ce/Cu diffusion layer.

## 4. Conclusions

In conclusion, a proper deposition temperature at 600 °C and a thinner film thickness of 100 or 200 nm facilitate the inhibition of CeFe_2_ phase in Ce-rich Nd_6.19_Ce_7.98_Fe_75.48_B_10.35_ (at.%) films. The changing deposition temperature and film thickness involves different CeFe_2_ contents. This has the advantage of allowing one to resolve a new decomposition process of Nd-Ce-Fe-B phase. Here, it was found that the unstable (Nd,Ce)_2_Fe_14_B phase is easily decomposed into the CeFe_2_ phase, concomitantly promoting the precipitation of the soft α-Fe phase. It is the soft-magnetic α-Fe induced by forming the CeFe_2_ phase that actually worsens kinks in the hysteresis loops usually observed in Ce-rich Nd-Ce-Fe-B magnets.

According to our studies, more CeFe_2_ phase will eventually worsen the kinks in hysteresis loops. It will promote the precipitation of soft α-Fe phase, which is also not beneficial for the improvement of coercivity. Thus, CeFe_2_ phase should not be formed too much when designing Ce-rich magnets. To acquire higher coercivity, other grain boundary phase addition should be explored. The coercivity in Nd_6.19_Ce_7.98_Fe_75.48_B_10.35_ (at.%) films can be significantly increased by about 4.9–9.1 kOe after addition of Ce-Cu diffusion layers without introducing much more worse kinks in the hysteresis loops. Our results are expected to provide important references for understanding the magnetic mechanisms in Ce-rich magnets and designing high-performance Ce-rich magnets.

## Figures and Tables

**Table 1:** The ratio of α-Fe to (Nd,Ce)_2_Fe_14_B in Ta/Nd-Ce-Fe-B/Ta films under different preparation condition.

Films	550 °C	600 °C	625 °C	650 °C	100 nm	200 nm	300 nm
x/y	28.7%	18.6%	27.8%	31.3%	11.8%	11.0%	41.6%

## Data Availability

The data presented in this study are available on request from the corresponding author.
